# Cell-Autonomous Death of Cerebellar Purkinje Neurons with Autophagy in Niemann-Pick Type C Disease

**DOI:** 10.1371/journal.pgen.0010007

**Published:** 2005-07-25

**Authors:** Dennis C Ko, Ljiljana Milenkovic, Steven M Beier, Hermogenes Manuel, JoAnn Buchanan, Matthew P Scott

**Affiliations:** Departments of Developmental Biology, Genetics, and Bioengineering, Howard Hughes Medical Institute, Stanford University School of Medicine, Stanford, California, United States of America; University of Minnesota, United States of America

## Abstract

Niemann-Pick type C is a neurodegenerative lysosomal storage disorder caused by mutations in either of two genes, *npc1* and *npc2*. Cells lacking Npc1, which is a transmembrane protein related to the Hedgehog receptor Patched, or Npc2, which is a secreted cholesterol-binding protein, have aberrant organelle trafficking and accumulate large quantities of cholesterol and other lipids. Though the Npc proteins are produced by all cells, cerebellar Purkinje neurons are especially sensitive to loss of Npc function. Since Niemann-Pick type C disease involves circulating molecules such as sterols and steroids and a robust inflammatory response within the brain parenchyma, it is crucial to determine whether external factors affect the survival of Purkinje cells (PCs). We investigated the basis of neurodegeneration in chimeric mice that have functional *npc1* in only some cells. Death of mutant *npc1* cells was not prevented by neighboring wild-type cells, and wild-type PCs were not poisoned by surrounding mutant *npc1* cells. PCs undergoing cell-autonomous degeneration have features consistent with autophagic cell death. Chimeric mice exhibited a remarkable delay and reduction of wasting and ataxia despite their substantial amount of mutant tissue and dying cells, revealing a robust mechanism that partially compensates for massive PC death.

## Synopsis

Niemann-Pick disease type C is a deadly neurodegenerative disease that is most often due to mutations in a gene called *npc1*. As a consequence of intracellular lipid trafficking defects, patients with Niemann-Pick type C, and mice with the same disease, lose an important class of cerebellar neurons called Purkinje cells (PCs). Npc1 (the protein coded by *npc1*) might be needed in other cell types to produce substances that nourish PCs or within the PCs themselves. To see which is true, the researchers constructed genetically mosaic mice in which some cells have mutant Npc1 and some have normal Npc1 function. In the cerebella of these mosaic mice, PCs lacking Npc1 continued to die even while surrounded by normal cells, while normal PCs appeared unaffected by their partially mutant surroundings. From these findings, the researchers concluded that the neurodegeneration is due to a problem within PCs and not due to a lack of supporting factors provided by other cells or an extrinsic toxic or inflammatory insult. Npc1 probably functions within PCs to allow critical transport processes necessary for cell survival. The researchers also found that the degenerating PCs undergo a complex process called autophagy in which the cells sense a lack of key nutrients and start to break down their own structures to feed themselves. By identifying exactly which cells require Npc1 function, the researchers set the stage for investigating the exact molecular roles of Npc1 protein in the cells where it is most needed.

## Introduction

Niemann-Pick type C (NPC) is a devastating autosomal recessive neurodegenerative disorder characterized by the accumulation of cholesterol and other lipids in viscera and the central nervous system. The clinical presentation typically includes progressive ataxia, dystonia, and dementia, first presenting in early childhood and ultimately leading to death in the early teens [1]. NPC is one of over 40 known lysosomal storage disorders (reviewed in [2,3]), which collectively have an incidence of one in 8,000 live births [4]. The specific molecules that accumulate in each disease vary, but most of the disorders share the feature of prominent neurological symptoms. Though great progress has been made in characterizing the biochemical and genetic defects in these diseases, the pathways that lead from these defects to cell and tissue dysfunction are inadequately understood.

The decline in neurological function in NPC patients is caused by mutations in either the *npc1* or *npc2* gene [5–7]. Npc1 is a multiple-membrane-spanning late endosomal/lysosomal protein that can bind cholesterol [8] and acts as a transmembrane pump when expressed in bacteria [9]. The Npc1 protein has a sequence related to the “sterol sensing domain” of known regulators of cholesterol metabolism (SCAP and HMG-CoA reductase) and Patched, a receptor for the secreted developmental signaling protein Hedgehog. The exact role of Npc1 in regulating lipid homeostasis and in maintaining neurological function is far from clear. Even less is known about Npc2, the small cholesterol-binding protein [10–12] responsible for a minority (<5%) of NPC cases.

Phenotypes similar to human NPC are seen in two mouse strains, C57BLKS/J *spm* [13] and BALB/c *npc1^nih^* [14], both of which harbor spontaneous mutations in *npc1* [6]. The most striking and well-documented histological change in *npc1* mice is the progressive loss of cerebellar Purkinje cells (PCs) [15,16]. Stereotactic cell counting confirmed that the PC is the type that exhibits the greatest percentage loss in *npc1* mice (<10% remaining at 10 wk) but surprisingly revealed that glia in the corpus callosum have a greater early reduction in number (48% for glia versus 13% for PCs, at 3 wk) [17]. This early decline in glia and the detection of Npc1 protein in astrocytic processes [18] led to the idea that the primary function of the Npc1 protein may be in glia and that loss of PCs and other neurons may be a secondary consequence of glial dysfunction [17,19]. However, Npc1 mRNA is abundantly present in neurons as well as glia throughout the brain [20]. Thus, it is unclear which cells in the brain require Npc1 protein and why PCs degenerate in mice lacking Npc1.

**Figure 1:**
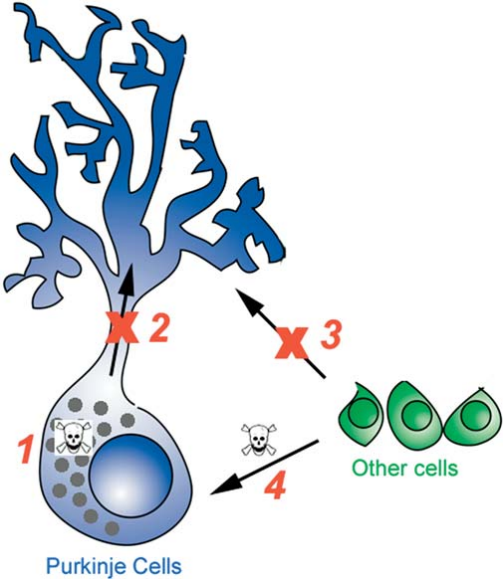
Models of PC Degeneration in *npc1* Mice Four basic mechanisms are depicted. (1) The accumulation of cholesterol, sphingolipids, or other molecules within PCs lacking Npc1 could be toxic. (2) Loss of Npc1 function could cause a block in trafficking that leads to a localized subcellular deficiency in lipids and/or proteins. (3) Other cells, most likely glia, could produce a secreted factor, such as apoE-cholesterol or a trophic factor required for PC survival, whose export is reduced or blocked without Npc1 function. (4) Any cell type in the *npc1* mouse could produce toxic metabolites, such as released lysosomal hydrolases or beta amyloid, that could kill surrounding cells or particularly susceptible cell types regardless of genotype.

Four broad categories of possible explanations for the PC degeneration have emerged (Figure 1). (1) Npc1 is required within PCs and loss of the protein results in degeneration as a consequence of the toxic accumulation of lipids. High concentrations of unesterified cholesterol can cause cell death by altering membrane rigidity, forming intracellular crystals that may interfere with organelle function, or triggering apoptotic signaling events [21]. (2) Npc1 is required within PCs and loss of the protein causes a deficiency at specific subcellular locations. In this model, the accumulation of cholesterol and other lipids is not harmful per se, but the diminished capacity to transport these or other molecules to the correct places within the cell leads to degeneration. (3) Npc1 is required within glia or another cell type within the cerebellum, or in a distant organ such as a secretory gland, and loss of the protein results in degeneration of PCs through a deficiency in a secreted molecule. This could be a trophic factor or possibly even cholesterol itself, as studies have shown that glia-derived cholesterol is required for synaptogenesis [22]. (4) Loss of Npc1 function results in the release of toxic/pro-inflammatory compounds into the local environment or systemic circulation, causing PC degeneration.

In this study, we employed chimeric mice to help resolve the fundamental mystery of why neurons degenerate in NPC disease. This powerful approach can distinguish intrinsic and extrinsic causes of cell death but has only rarely been used to study human neurodegenerative disease [23,24]. The results indicate that PC loss in *npc1* ^−/−^ mutants is a cell-autonomous process, i.e., Npc1 function is critical within PCs. Ultrastructural and biochemical analyses support the idea that this cell-autonomous loss is due to the activation of a particular genetic program, autophagy, that leads to cell death.

## Results

### Pattern of Cerebellar Degeneration in *npc1* Mice

**Figure 2:**
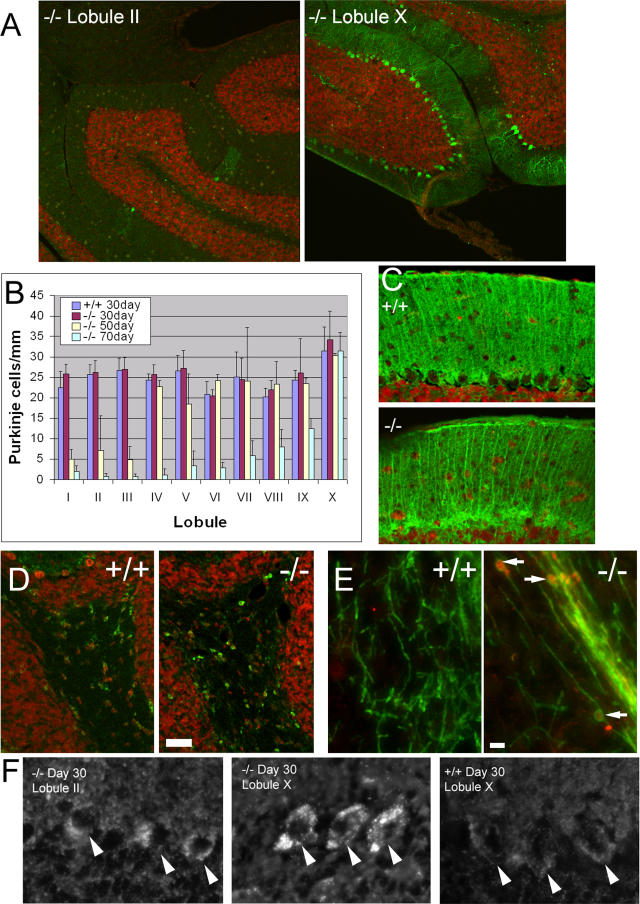
Features of PC Loss in *npc1* Mice (A) Sections from the cerebellar vermis of a 70-d-old *npc1^−/−^* mouse were stained with anti-Calbindin (green) to visualize PCs and 7AAD (red) for nuclei. Although there are scant PCs remaining in lobule II, the PCs in lobule X have not decreased. (B) Quantification of progressive anterior-to-posterior PC loss. PC densities from 30-d *npc1* ^+/+^ and 30-, 50-, and 70-d *npc1* ^−/−^ mice were quantified from five sections from two mice each. Error bars show standard deviation. (C) Bergmann glia visualized in 70-d *npc1* ^+/+^ and *npc1* ^−/−^ mice using anti-S100β (green) have normal morphology. Note that the glial cell bodies near the PC layer and the radial processes extending into the molecular layer are intact in the *npc1* ^−/−^ mouse despite the loss of PCs. (D) Oligodendrocyte cell bodies stained with anti-CC1 (green) have a similar distribution and morphology in the cerebellar white matter of 70-d *npc1^−/−^* and wild-type mice. TOTO-3 (red) was used as a nuclear counterstain. Bar = 5 μm. (E) Axonal spheroids in *npc1* ^−/−^ mice. Spheroids (arrows), seen as Calbindin-positive (red) swellings surrounded by myelin (anti-myelin basic protein; green), are numerous in the *npc1^−/−^* cerebellum (50-d-old, lobule X shown) but are not seen in the wild-type control. Bar = 5 μm. (F) Sections from wild-type and *npc1* mice were stained with filipin to visualize free cholesterol. PCs from *npc1* mice show an accumulation of intracellular cholesterol at 30 d regardless of their lobular location. Images are oriented such that the PCs (arrowheads) are in the center of the field, with the molecular layer above and the inner granule cell layer below.

As a first step it was important to observe and measure progressive PC loss. PC loss in the cerebellar vermis progressed in an anterior (lobule I) to posterior (lobule X) sweep, as previously reported [15,16] (Figure 2A and 2B). There was a normal number and density of PCs in cerebellar sections from 30-d-old *npc1^−/−^* mice. At 50 d, most PCs in lobules I–III were no longer present. By 70 d, only lobule X PCs remained intact.

We next investigated whether Bergmann glia are affected in *npc1^−/−^* mice. Bergmann glia appose the PC soma and extend processes into the molecular layer that sheaths the PC dendritic trees (reviewed in [25]). Loss or dysfunction of these glia could precede and precipitate the PC loss. In contrast to the degeneration and loss of PCs, Bergmann glia stained with anti-S100β maintain their density and their characteristic radial morphology, even at 70 d, in all lobules (Figure 2C). Oligodendrocytes, the glia that myelinate axons, were normal in distribution and cell body morphology (Figure 2D). Staining of myelin revealed axonal spheroids as previously reported (Figure 2E) [15,26]. Granule cells, the most abundant neurons in the cerebellum, were also grossly normal based on anti-GABAα6 staining (data not shown). This indicates that loss of PCs in *npc1* mice is not due secondarily to a loss or other obvious morphological derangement of these other cell types. However, these observations do not rule out the possibility that a more subtle dysfunction in these cells contributes to the degeneration of PCs in *npc1*mice.

The anterior-to-posterior gradient of PC loss provides the opportunity to determine how cholesterol accumulation changes in relation to the PCs. If intracellular cholesterol accumulation is toxic to PCs, a correlation is expected between the amount of cholesterol and the rate or severity of degeneration. Instead, we find that at 30 d, intracellular cholesterol accumulation is noted even in lobule X PCs (which do not degenerate during the normal *npc1* mouse life span). We did not find any consistent difference in filipin staining of cholesterol among the different cerebellar lobules in *npc1* mice (Figure 2F). Therefore, lysosomal cholesterol accumulation per se is not sufficient to explain the pattern of PC death.

### PC Degeneration in *npc1* Mice Is Cell-Autonomous

**Figure 3:**
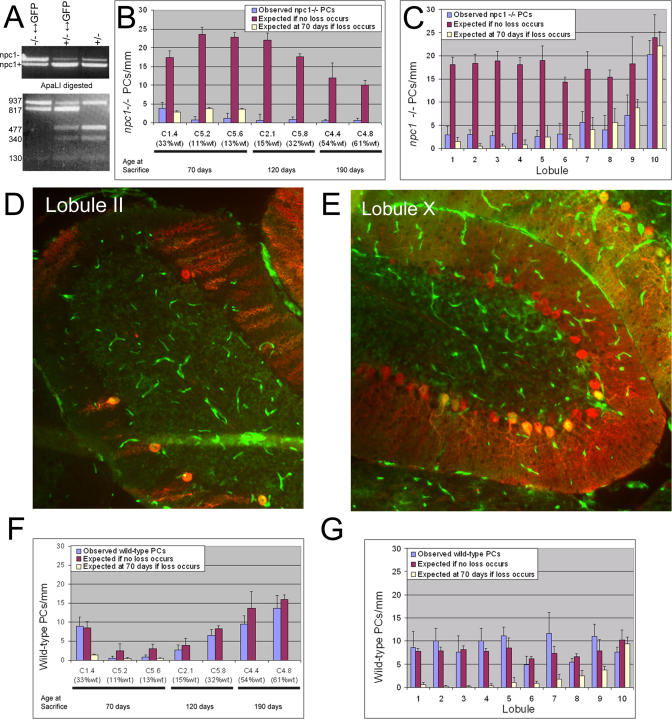
Cell-Autonomous Degeneration of PCs in Chimeric Mice (A) Genotyping of chimeric mice. Upper gel: PCR to amplify a fragment of the *npc1* gene from *npc1* ^−/−^↔GFP, *npc1* ^+/−^↔GFP, and *npc1* ^+/−^mice results in *npc1^−^* (1,067 bp) and *npc1^+^* (947 bp) bands. Lower gel: The three genotypes can be distinguished by ApaLI digestion of the PCR products. The *npc1^−^* allele gives rise to 937- and 130-bp bands. The wild-type allele varies depending on the source: the BALB/c allele is digested to 477-, 340-, and 130-bp bands while the allele from the GFP mouse is digested to 817- and 130-bp bands. Note that the 477- and 340-bp bands are absent from the homozygous mutant chimera. (B) Quantification of *npc1* ^−/−^ PC density in chimeric mice. For each mouse, the actual density observed (the average of the mean densities counted in each lobule) is compared to the number expected if no PC loss occurs (calculated from the density at 30 d in *npc1* ^−/−^ mice and the chimerism percentage). Five sections separated by at least 100 μm were analyzed for each mouse. For the three mice sacrificed at 70 d, only lobules I–IX were included in the analysis because of the lack of degeneration in lobule X in *npc1* ^−/−^ mice at this age. A value for the number of *npc1* ^−/−^ PCs expected if loss does occur (calculated from the density at 70 d in *npc1* ^−/−^ mice and the chimerism percentage) is also included for these three mice. No rescue of *npc1* ^−/−^ PCs was observed for any of the chimeric mice (*p* < 0.0001 for each mouse comparing observed versus expected if no loss occurs). (C) Quantification of *npc1^−/−^* PC density by lobule in C1.4 at 70 d. Observed and expected densities are shown for *npc1* ^−/−^ PCs in each lobule. No clear rescue of *npc1* ^−/−^ PCs was observed in any lobule (*p* > 0.05 for all lobules comparing observed versus expected if loss occurs, except II and III, where *p*-value is between 0.01 and 0.05; *p* < 0.001 for all lobules comparing observed versus expected if no loss occurs, except lobule X, where *p* > 0.05). (D and E) Images of lobules II and X taken from C1.4 at 70 d. GFP is green and Calbindin staining is red. In lobule II, the number of PCs is clearly reduced and of the five remaining PCs, four are wild-type (GFP-positive). In lobule X (where no degeneration occurs during the normal *npc1* ^−/−^ life span), PC density is normal and the majority of PCs (23 of 32; 72%) are mutant, as expected based on *npc1* ^−/−^ contribution to this mouse (67%). (F) Quantification of wild-type PC density in chimeric mice. Observed and expected densities are shown for wild-type PCs. For mice with more than 15% wild-type contribution, the density of wild-type PCs is similar to the number expected if these cells have not degenerated (*p* > 0.05 for each mouse comparing observed versus expected if no loss occurs, except C5.2, where *p* = 0.02, and C5.6, where *p* = 0.0005). (G) Quantification of wild-type PC density by lobules in C1.4 at 70 d. No loss of wild-type PCs was observed in any of the lobules (*p* > 0.05 for all lobules comparing observed versus expected if no loss occurs; *p* < 0.001 for all lobules comparing observed versus expected if loss occurs, except for lobule X, where *p* > 0.05).

To determine whether PC loss is due to dysfunction within the PCs, alterations in the surrounding glia and microenvironment, or a combination of both, we generated mice containing a mixture of *npc1* mutant and wild-type cells. Morulas derived from mating *npc1* heterozygotes (BALB/c *npc^nih^/+*) and from matings of wild-type mice homozygous for a ubiquitously expressed green fluorescent protein (GFP) gene (FVB.Cg-Tg(GFPU)5Nagy/J; [27]) were aggregated to form chimeric embryos. A quarter of the embryos from *npc1/+* parents should be homozygous for the *npc1* mutation, but these embryos could not be distinguished at the time of morula isolation, so the embryos derived from aggregation had to be analyzed later to determine which were the useful chimeras. Chimeras were also created in which *npc1* mutant cells were marked with GFP (genetic background 75% FVB/25% BALB/c; see Materials and Methods for crosses), ensuring that the results were independent of the genetic background. Of 33 chimeric mice generated, seven were of the desired genotype, as determined using PCR analysis (two *npc1* ^−/−^ BALB/c↔GFP; two *npc1* ^−/−^ 75% FVB/25% BALB/c↔CD-1; three *npcl* ^−/−^ 75%FVB/25% BALB/c↔C57BL6/J) (Figure 3A). These seven mice had wild-type contributions ranging from 11% to 61% based on skin fluorescence, granule cell counts, and lobule X PC counts. Wild-type contributions estimated from skin fluorescence always corresponded well to the value determined from cell counts; the numbers differed by 4%–22% (see Table 1). The reliability of skin fluorescence and granule cell counts as estimates of overall chimerism percentage was further validated by examination of two *npc1* heterozygous chimeras that demonstrated similar chimerism percentage whether determined by skin, granule cell counts, or, most importantly, PC counts (Table 1). For each chimeric mouse, all PCs in five sagittal sections of the cerebellar vermis were counted and each cell's genotype was determined based on GFP fluorescence. Characteristics of the mice are summarized in Table 1.

**Table 1:**
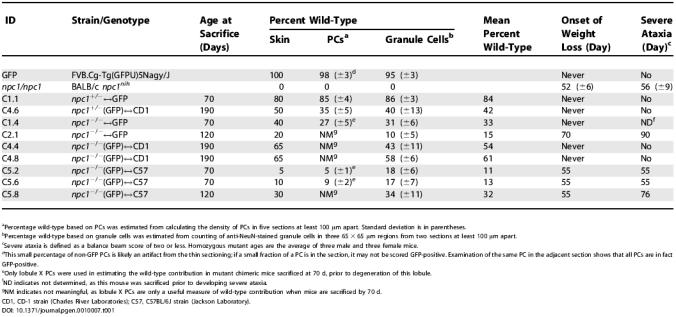
Genotype and Percentage Chimerism for the Mice Used in This Study

^a^Percentage wild-type based on PCs was estimated from calculating the density of PCs in five sections at least 100 μm apart. Standard deviation is in parentheses.

^b^Percentage wild-type based on granule cells was estimated from counting of anti-NeuN-stained granule cells in three 65 × 65 μm regions from two sections at least 100 μm apart.

^c^Severe ataxia is defined as a balance beam score of two or less. Homozygous mutant ages are the average of three male and three female mice.

^d^This small percentage of non-GFP PCs is likely an artifact from the thin sectioning; if a small fraction of a PC is in the section, it may not be scored GFP-positive. Examination of the same PC in the adjacent section shows that all PCs are in fact GFP-positive.

^e^Only lobule X PCs were used in estimating the wild-type contribution in mutant chimeric mice sacrificed at 70 d, prior to degeneration of this lobule.

^f^ND indicates not determined, as this mouse was sacrificed prior to developing severe ataxia.

^g^NM indicates not meaningful, as lobule X PCs are only a useful measure of wild-type contribution when mice are sacrificed by 70 d.

CD1, CD-1 strain (Charles River Laboratories); C57, C57BL/6J strain (Jackson Laboratory).

The data from all seven mice demonstrated that PC degeneration in *npc1^−/−^* mice is primarily a cell-autonomous process. PC counts revealed that the presence of a milieu that was more than 60% wild-type was insufficient to prevent *npc1* PC loss. In the two oldest mice analyzed (190 d), chimeras 4.4 and 4.8 (C4.4 and C4.8), nearly all *npc1* PCs had degenerated, including those in lobule X. In these mice, the wild-type contributions were estimated to be 54% and 61%. Mutant *npc1* PCs were observed at densities of 0.5 and 0.6 PCs/mm in the mice, strikingly lower than the expected densities of 12 and 10 PCs/mm if no degeneration had occurred (Figure 3B). Expected PC densities, assuming no degeneration, were calculated by multiplying the observed density in 30 d *npc1^−/−^* mice by the percentage of chimerism. Similar measurements were made in all seven chimeras. Two mice sacrificed at a younger age (120 d; C2.1 and C5.8) had lost all but a few *npc1* PCs, which were primarily in lobule X. The results demonstrate that surrounding wild-type cells are unable to prevent *npc1* PC degeneration. These four mice also demonstrate that lobule X PCs will eventually degenerate; their loss is delayed rather than completely prevented.

Three chimeric mice were analyzed at 70 d, at the end of the normal life span of *npc1* ^−/−^ mice. One of these three mice had a substantial wild-type contribution (33%; C1.4), but no significant prevention or delay of *npc1* PC degeneration was observed (Figure 3C). The vast majority of mutant PCs (except those in lobule X) degenerated despite the presence of a significant fraction of wild-type cells (Figure 3D and 3E).

In contrast to the mutant PCs, wild-type PCs did not degenerate in most of the chimeric mice. The observed wild-type PC densities agreed well with the expected densities based on the wild-type contribution in each mouse (Figures 3F and 3G). The lone exceptions occurred in the two mice with 11% and 13% wild-type contribution. These mice were expected to have a very low number of wild-type PCs, but there were even fewer than expected. No significant decrease in wild-type PC density was observed in the other five chimeric mice, including C2.1, which was expected to have only a marginally greater wild-type contribution (15%). Although fluctuation in how small numbers of wild-type cells are distributed and consequent variability among analyzed sections provide a likely explanation, another interpretation is that when very few wild-type cells are present, the entire cerebellum begins to break down during the late stages of cerebellar degeneration. Toxins released by dying cells could contribute to PC loss but are clearly not the main cause of neurodegeneration.

### Other Histologic Features of *npc1* Chimeric Mice

**Figure 4:**
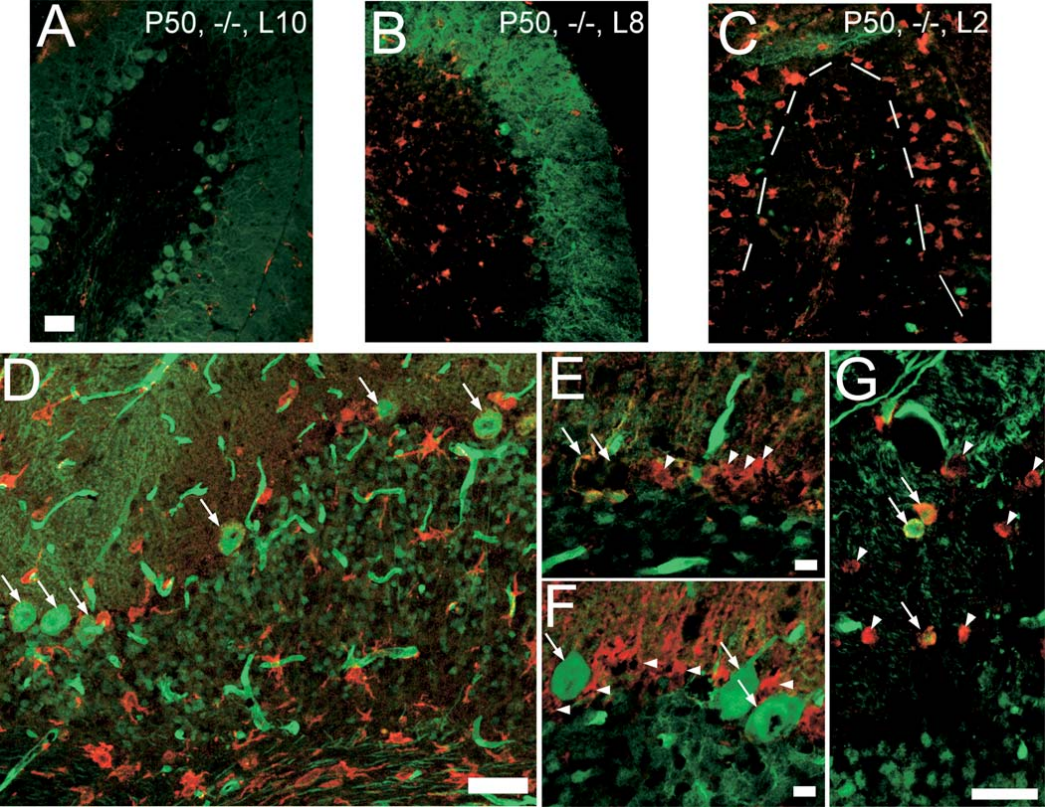
Other Histological Characteristics in the *npc1* Mutant and Chimeric Mice (A–C) Microglia characteristics. Sections from 50-d-old *npc1* mutants were stained with anti-Calbindin (green) to visualize PCs and anti-F4/80 (red) to mark microglia. (A) Very few microglia are present in lobule X, where no PC degeneration has yet occurred. (B) Lobule VIII demonstrates an infiltration of microglia in the granule cell layer and white matter tract. (C) In lobule II, nearly all PCs are gone and numerous microglia are present throughout the cerebellum. The dashed line indicates the edge of the granule cell layer. (D) Microglia infiltration is not sufficient to induce PC degeneration. C1.4 demonstrates numerous microglia marked by anti-F4/80 (red) in all layers of the cerebellum, including immediately adjacent to wild-type (GFP-positive) PCs (arrows). Despite this close approximation, no loss of wild-type PCs was detected by cell counting. The GFP transgene is apparently poorly expressed in microglia, as all microglia appear GFP-negative regardless of their genotype. (E) Mutant PCs are lost even when surrounded by wild-type Bergmann glia. A stretch of the PC layer in C4.8 is shown where no *npc1^−/−^* (GFP-positive) PCs remain despite the presence of numerous wild-type (GFP-negative) glia (arrowheads) marked by anti-S100β (red). The space occupied by two wild-type PCs is indicated (arrows) with two *npc1^−/−^* (GFP-positive) glia directly underneath. (F) Wild-type PCs do not degenerate even when surrounded by mutant Bergmann glia. Three wild-type PCs (GFP-positive; arrows) in C1.4 have not degenerated despite only mutant glia (arrowheads) being in the immediate vicinity. (G) Oligodendrocytes in chimeric mice are a mixture of wild-type and mutant cells. Oligodendrocyte cell bodies are stained with anti-CC1 (red). Wild-type (GFP-positive; arrows) and *npc1^−/−^* (GFP-negative; arrowheads) oligodendrocytes are interspersed in C1.4. Bar = 50 μm for (A), (D), and (G); 10 μm for (E) and (F).

Examination of the relationships of other cell types to the PCs in the chimeras provides further evidence for the cell-autonomous nature of PC degeneration. Microglia have been observed in the cerebellum of *npc1^−/−^* mice, but their role in PC degeneration is unclear [28]. Microglia are the phagocytic cells of the central nervous system, and their presence is indicative of an inflammatory process. The appearance of microglia within the cerebellum follows the anterior–posterior pattern of PC death. In wild-type mice or *npc1* mutant mice at 30 d, almost no microglia are observed (data not shown). In 50-d-old *npc1* mutant mice, very few microglia are present in lobule X (Figure 4A). The other cerebellar lobules have numerous microglia in the white matter tract and granule cell layer prior to the loss of PCs (Figure 4B). After more extensive PC loss (lobules I–IV at 50 d and I–IX at 70 d), microglia also appear in the molecular and PC layers (Figure 4C). Thus, the arrival of microglia coincides with PC loss. However, the infiltration of numerous microglia is not sufficient to trigger PC degeneration. Microglia were present in all the chimeric mice (though to a lesser extent in mice sacrificed at a later age) and were in close proximity to wild-type PCs (Figure 4D, white arrows). Despite this close association, there was no decrease in wild-type PC density in the chimeras except the two noted above. Furthermore, if nonspecific microglial killing of PCs were the main mechanism of PC loss, then wild-type and mutant PCs should demonstrate comparable decline percentages. This is not the case in any of the chimeras.

Mutant PC loss occurred even with wild-type Bergmann glia in the immediate vicinity. In the chimeras, PCs were strikingly absent in regions containing abundant wild-type glia (Figure 4E, arrowheads). Conversely, wild-type PCs (Figure 4F, white arrows) survived even when surrounded by mutant glia (Figure 4F, arrowheads). Oligodendrocytes of both genotypes were scattered in the cerebellar white matter of the chimeric mice (Figure 4G). The loss of mutant PCs is unlikely to be due to the lack of a secreted factor from supporting cells. If Npc1 were necessary for the efficient secretion of this hypothetical survival factor, then surrounding wild-type glia should have been able to provide the molecule to mutant PCs. The ongoing degeneration of *npc1* mutant PCs surrounded by a high percentage of wild-type cells indicates that cell-autonomous mechanisms play the primary role in PC death.

### Prevention of Ataxia and Weight Loss in Chimeric Mice

**Figure 5:**
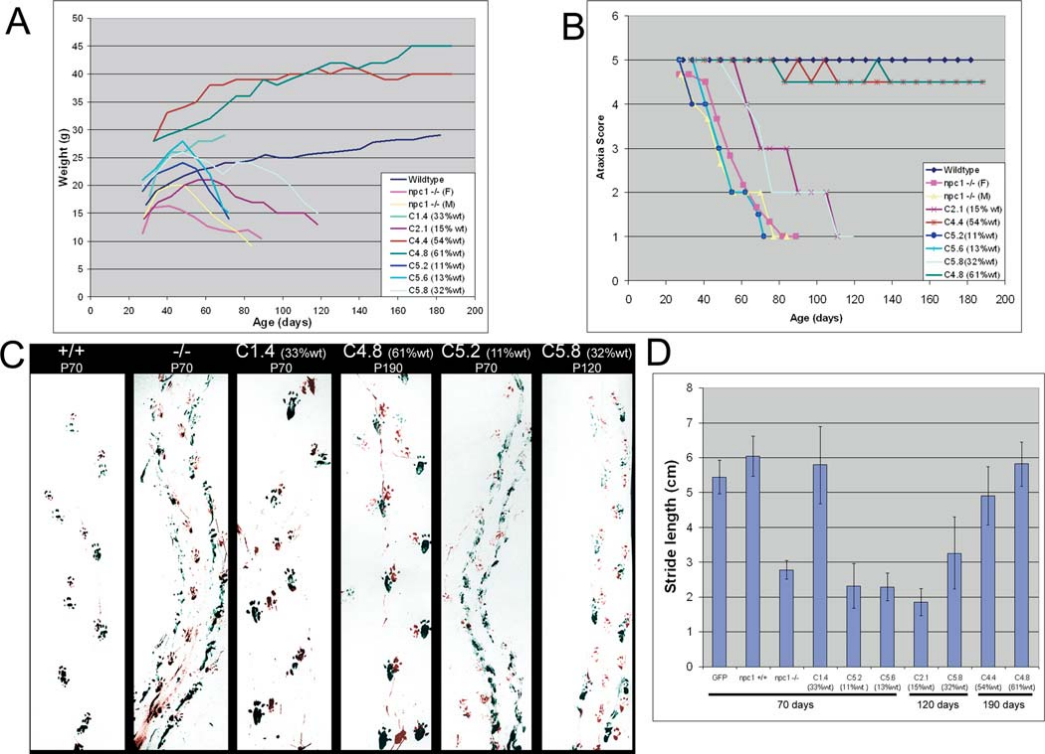
Organismal Phenotypes in Chimeric Mice (A) Chimeric mice with more than 50% wild-type contribution do not exhibit wasting. Weekly weights for each of the mice were recorded from 4 wk until the mice were sacrificed. For wild-type and *npc1^−/−^* homozygous mice, the curve shown is the mean weight from three to four mice. A progressive decrease in weight of *npc1* mice begins to show around 7 wk. The chimeric mice display a delay in, or even absence of, wasting depending on the amount of wild-type contribution. (B) Prevention of ataxia in chimeric mice. Mice were assessed for ataxia weekly during two 3-min trials on a balance beam (see Materials and Methods). (C) Gait of chimeric mice. Front and back paws of mice were dipped in red or green paint, and mice walked across a box lined with paper. The *npc1* ^−/−^ mouse displays shorter stride length and a smearing of the footprints as the paws are not as well lifted between steps. Chimeric mice with high amounts of wild-type contribution (C4.8; C4.4 not shown) exhibited a gait indistinguishable from wild-type. (D) Quantification of stride length. For wild-type and mutant controls, three mice were assessed. For chimeric mice, gait was measured prior to sacrifice at the ages noted. Error bars indicate the standard deviation. Comparison with the GFP mice demonstrates that *npc1^+/+^*, C1.4, C4.4, and C4.8 do not have significantly different stride lengths (*p* > 0.05) while *npc1^−/−^*, C5.2, C5.6, C2.1, and C5.8 have significantly shorter stride lengths (*p* < 0.001)

Despite substantial loss of *npc1* PCs, some chimeric mice displayed minimal ataxia and no weight loss. Ataxia was assessed through measuring agility on a balance beam [29] and by quantifying step size in a gait assay [30]. At 190 d, more than twice the normal life span of *npc1* mice, the two chimeras with more than 50% wild-type cells (C4.4 and C4.8) displayed no signs of wasting and only minimal ataxia (Figure 5). Even more impressive was a mouse with only 15% wild-type cells (C2.1) that had significantly reduced symptoms. The genetic background of the mutant cells appears to influence the rate of disease progression. Delayed symptoms were not noted in C5.2 and C5.6, which had only slightly less wild-type contribution than C2.1. The *npc1* mutant cells in the C5.2 and C5.6 mice were derived from a 75% FVB/25% BALB/c↔GFP^+/+^ background, and mutant mice of this genetic background demonstrated a faster progression of disease compared to the mutation in the BALB/c background of C2.1 (average life span of 67 ± 5 d versus 81 ± 8 d). It is remarkable that some 40% of the PCs can die without leading to severe ataxia or weight loss, and that even much smaller numbers of wild-type cells (15%) provide temporary protection against wasting, ataxia, and death.

### Activation of Autophagy in Degenerating PCs

The evidence from chimeric mice highlighted the importance of possible cell-autonomous mechanisms of PC degeneration. Necrosis, apoptosis, and autophagy of PCs all occur in mouse models of neurodegeneration (reviewed in [31]). The cell death mechanism at work in NPC is unknown, although a very recent report demonstrated TUNEL staining and increased caspase-8 levels in *npc1* ^−/−^ cerebellum, supporting a role for apoptosis in neurodegeneration in NPC [32]. We addressed this question by ultrastructural comparison of wild-type and *npc1* PCs using electron microscopy (EM). No apoptotic bodies were identified by EM, nor did we identify any TUNEL-positive PCs using immunofluorescence labeling of sections from 48-d-old *npc1^−/−^* mice (data not shown).

**Figure 6:**
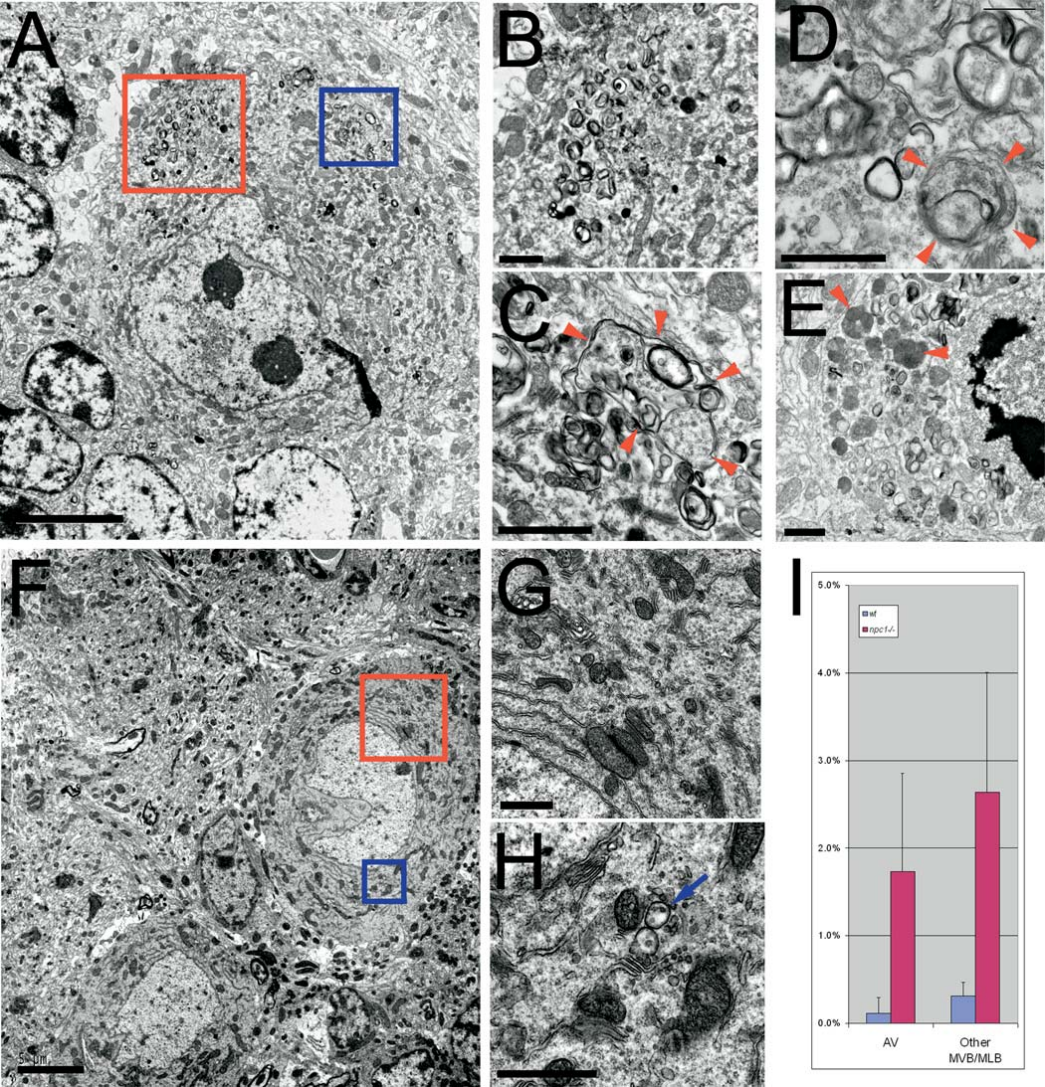
Increased Autophagy in *npc1* PCs EM of *npc1^−/−^* and wild-type PCs from anterior vermis of 48-d-old mice. (A) Low-power field of a typical *npc1* PC. (B) Magnification of the red box in (A) showing accumulated multivesicular and multilamellar organelles. (C) Magnification of the blue box in (A) showing a large AV (arrowheads) containing ribosomes and membranes. (D) Another AV (arrowhead) with ER cisternae within the lumen. (E) An *npc1* PC probably in the late stages of degeneration, with many AVs, some with apparently degrading mitochondria (arrowheads). (F) Low-power field of two wild-type PCs. (G and H) Magnification of the red (G) and blue (H) boxes in (F) showing no AVs and very few multivesicular organelles (arrow). (I) Quantification of area occupied by AVs and other multivesicular/multilamellar organelles expressed as a percentage of total cytoplasmic area for seven wild-type and seven *npc1^−/−^* PCs; *p* = 0.0009 for comparing AV areas and *p* = 0.0003 for comparing other multivesicular/multilamellar areas, indicating both types of organelles occupy significantly more area in *npc1^−/−^* PCs. Bar = 5 μm for (A) and (F); for all others, bar = 1 μm.

In contrast, numerous structures with features consistent with autophagic vacuoles (AVs) were identified in *npc1* PCs by EM. Previous EM studies of *npc1* PCs [13,16] noted the appearance of “lamellar inclusion bodies.” Such structures are consistent with the activation of autophagy, although to our knowledge there have been no reports of autophagy in NPC. AVs can be classified as autophagosomes or autolysosomes based on morphological criteria [33,34]. Autophagosomes are bound by two or more membranes and contain seemingly unaltered cytoplasmic components. The autophagic and endocytic pathways converge following fusion of autophagosomes with late endosomes and subsequently with lysosomes [35]. These fusion events result in autolysosomes, organelles enclosed by a single limiting membrane with degrading membranes and cellular components within. Numerous AVs are found in PCs from 48-d-old *npc1* homozygous mice (Figure 6A–6E). Such structures are rarely seen in 48-d-old wild-type mice (Figure 6F–6H). Since NPC cells accumulate lipids within organelles containing late endocytic and lysosomal markers, the structures we identified as AVs could be aberrant late endosomes or lysosomes, with no autophagy involved. However, we can clearly identify cytoplasmic contents (free ribosomes, endoplasmic reticulum [ER] cisternae, and mitochondria) within these structures, a diagnostic for AVs. Quantification of AVs in wild-type and *npc1* PCs demonstrates that these structures occupy 15 times more area in *npc1* PCs (Figure 6I). Rarely, the cell body of an *npc1* PC filled up almost completely with autolysosomes (Figure 6E). Degenerating PCs in *npc1* mice clearly have morphological features consistent with autophagic cell death.

**Figure 7:**
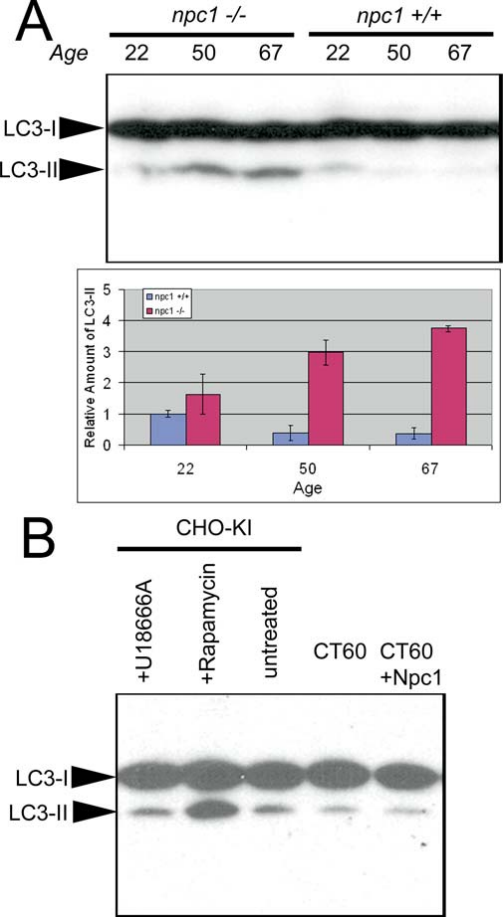
Levels of the Autophagosomal Marker LC3-II Are Increased in Degenerating *npc1* Cerebellum (A) Immunoblots of 70 μg of protein resolved by 15% SDS-PAGE show increased levels of LC3-II in 50- and 67-d-old *npc1* cerebellar extracts. Two mice of each age and genotype were analyzed, and relative band intensities were quantified. *p-*Values derived from comparing wild-type and *npc1^−/−^* cerebella at each age are 0.3, 0.01, and 0.001 for 22-, 50-, and 67-d-old mice, respectively. (B) Immunoblots of CHO cell extracts resolved by 15% SDS-PAGE do not show increased levels of LC3-II in CT60 cells compared to wild-type CHO-KI cells or CT60 cells stably transfected with NPC1-YFP. No difference is noted with 10 μM U18666A, a drug that mimics *npc1* loss of function. Rapamycin treatment (1 μM) of CHO-KI cells demonstrates robust activation of autophagy with increased LC3-II levels.

Increased modification of an autophagosomal marker protein provides further evidence for the activation of autophagy in *npc1* cerebellum. During autophagy induced by starvation or rapamycin, the light chain 3 (LC3) protein undergoes lipid modification by a ubiquitin-like conjugating system [36,37]. This lipid-modified form of the protein (LC3-II) associates with autophagosome membranes, and levels of LC3-II correlate with the amount of autophagosome formation [38]. In adult cerebellar cortex, LC3 protein is reported to be present primarily in PCs, based on immunofluorescence studies [39]. The amount of LC3-II protein in *npc1* ^−/−^ cerebellum increases dramatically with age (Figure 7A). When 22-d-old mice are compared, LC3-II levels are nearly equivalent. In wild-type mice, the level stays the same or may slightly decrease in 50- and 67-d-old mice. In the *npc1^−/−^* mice, there was a 7- and 11-fold increase in LC3-II compared to age-matched controls of these respective ages. Thus, morphological and biochemical criteria show that autophagy is activated in degenerating *npc1^−/−^* cerebella.

To determine whether loss of *npc1* is sufficient to induce autophagy in all cell types, we examined CT60 cells, a cell line harboring a loss-of-function mutation in *npc1* [40]. No elevation of LC3-II was detected, though LC3-II accumulation can clearly be induced in Chinese hamster ovary (CHO) cells with rapamycin treatment (Figure 7B). The lack of autophagy activation in an *npc1* cell line and in cerebella from 22-d-old *npc1^−/−^* mice suggests that autophagy is activated only in *npc1^−/−^* cells actively undergoing degeneration.

## Discussion

Neurodegeneration is among the most devastating and feared types of human disease. No treatments exist that stop neurodegeneration and very few even provide transient symptomatic relief [41]. Alzheimer disease, the most common cause of dementia, affects roughly 12 million people worldwide [42]. Even for Alzheimer disease the pathophysiology is unclear. It is still controversial whether extracellular or intracellular beta-amyloid deposits are responsible for the bulk of neurotoxicity [43,44], and the importance of inflammation to progression of the disease is unknown [45,46]. For most neurodegenerative diseases, how much neuronal loss can be attributed to cell-autonomous versus non-cell-autonomous factors is unknown. We examined this crucial question in the neurodegenerative disorder NPC.

People born with NPC disease undergo a progressive decline in neurological function that can include ataxia, tremor, dystonia, dementia, and seizures. As the prominent ataxia would suggest, cerebellar involvement, including PC degeneration, has been well documented [47–49]. The mouse model of NPC faithfully recapitulates many of the human symptoms, and PC loss serves as an easily quantifiable measure of neurodegeneration. The involvement of circulating molecules such as steroids and sterols, and the ubiquitous expression of both NPC genes, make determining when and where the NPC genes are required a key first step in understanding neurodegeneration. A previous study highlighted the importance of events within the central nervous system, as expression of Npc1 driven by the prion promoter could prevent neurodegeneration even without complete rescue of the liver phenotype [30]. In this study we have found that the most prominent neurodegenerative event, the death of the PCs, is due to lost Npc1 function within those cells and to a distinctive programmed cellular response, autophagy.

### Cell-Autonomous PC Death in NPC Disease

We have demonstrated that the loss of PCs in the mouse model of NPC, which has a mutant *npc1* gene, is primarily a cell-autonomous defect. This finding places constraints on possible mechanisms of PC degeneration in NPC disease, and allows future investigations to focus on the cell type that matters. As is described in the introduction, several lines of evidence hinted that neurodegeneration in NPC disease could be a non-cell-autonomous process. However, the inability of a wild-type milieu, including surrounding wild-type glia, to prevent *npc1^−/−^* PC loss indicates that the degeneration of PCs is not due to the loss of a secreted factor such as glia-derived sterols, neurotrophins, or Npc2 protein. Consistent with this, two very recent studies failed to detect a difference in levels of secretion of cholesterol from wild-type versus *npc1^−/−^* glia [50,51].

In addition to surrounding glial cells, the health of PCs could be affected by their axonal targets in the cerebellar deep nuclei. Expression of GFP is very low in neurons of the cerebellar deep nuclei in the GFP mice (data not shown), so we were unable to assess the genotype of these cells in the chimeras. During the migration of PCs, their axons do not remain attached to their future post-synaptic targets [52]. If PC death in *npc1* mice was caused by loss of a target-derived factor, then the absence of a genotypic match between target neurons and PCs would mean that wild-type and *npc1* PCs should be equally affected. This is not what we observe. In addition, completely depriving PCs of their axonal targets by axotomy does not cause PC degeneration even up to a year following axotomy [53,54]. Thus, derangement of cerebellar deep nuclei is unlikely to be causing PC degeneration in NPC.

Secreted or released toxic molecules are also not responsible for PC death. Microglia produce pro-inflammatory and cytotoxic compounds including cytokines, proteases, and free radicals [55–57] and are present in higher-than-normal numbers in the NPC mutant cerebellum [28]. A robust inflammatory response may play a role in the progressive neurodegeneration seen in NPC but is not the primary factor in causing neuronal loss: in the chimeric mice most wild-type PCs do not degenerate even while in the midst of numerous microglia. Our experiments do not exclude a more cell-specific role for microglia in PC death. Microglia could help trigger cell death by specific molecular interactions with susceptible PCs, as is proposed to occur during PC death normally seen at postnatal day 3 [58]. A microglia-based mechanism would require a cell-autonomous component—PCs expressing the proper membrane receptors—and a non-cell-autonomous component—microglia to carry out the directed engulfment of only those cells.

### Internal Factors in Neurodegeneration

PCs may degenerate either (1) because the buildup of sterols and other lipids, or other unknown substances, poisons the PCs or (2) because trafficking defects resulting from Npc1 loss affect cell survival. Although excess cholesterol can have toxic effects [21], intracellular cholesterol levels within PCs do not correlate with the loss of these cells: lobule X PCs accumulate cholesterol and do not degenerate during the 70 d that normally constitute the *npc1* mouse life span. More subtle differences among lobules, in sterol mass or subcellular localization of cholesterol, are unlikely to be detected with filipin staining. In any case, blocking lipid accumulation is insufficient to slow neuronal loss, again arguing against the idea that cell death occurs because of accumulated lipid. Crossing the *npc1* mouse to a GalNAcT-knockout mouse unable to synthesize complex gangliosides decreases levels of sphingolipids and cholesterol without any effect on neurodegeneration [59]. Crossing the *npc1* mouse to a low density lipoprotein receptor knockout also does not slow neurodegeneration [60]. Although both of these experiments have their weaknesses—possible buildup of less complex sphingolipids in the GalNAcT experiment and possible redundancy in lipoprotein receptors in the low density lipoprotein receptor knockout—the findings at least suggest that intracellular toxicity due to lipid accumulation is not the primary mechanism of PC death.

Defects in lipid trafficking are likely to have particularly detrimental effects on synthetic processes that rely on lipids as substrates. A recent study demonstrates that neurosteroid synthesis is reduced in *npc1* mice and administration of allopregnanolone increases life span and neuronal survival [61]. PC loss involving interference with production of neurosteroids acting in a paracrine or endocrine manner is untenable in light of our findings. An autocrine mechanism of action, with PCs making substances that act in the producing cell, is still plausible and needs to be explored further. PCs synthesize several different neurosteroids and express progesterone receptors [62]. PCs are unique in having an extensive smooth ER, the “hypolemmal cisternae,” that is “so well developed in PCs that it may be considered a specific characteristic…since it extends under the plasmalemma of the entire cell body, the dendritic tree, and the axon” [63]. Palay and Chan-Palay [63] also note that mitochondria are often found against the inner surface of the hypolemmal cisternae. Impaired cholesterol trafficking to mitochondria and the smooth ER may lead to a deficiency at these sites, lowering the production of neurosteroids.

### Autophagy in NPC PC Death

Loss of *npc1* function within PCs leads to increased autophagy and cell death. Apoptosis may also be involved in the death of neurons from NPC disease; both processes are likely at work in the degenerating NPC brain. In contrast to apoptosis, which has been demonstrated in many instances of neurodegeneration (reviewed in [31]), activated autophagy has been demonstrated in only a few cases, but notably including death of PCs in *Lurcher* mice [64]. The role of autophagy in neurodegenerative disease extends to Huntington disease and Parkinson disease [65–67]. These neurodegenerative diseases differ from NPC in that they are disorders of protein aggregation. Formation of insoluble aggregates of Huntingtin or alpha-synuclein is an early event in disease progression [68–71]. Autophagy may be activated by cells in order to dispose of the aggregates. Deliberately enhancing degradation of protein aggregates by triggering autophagy in animal models of Huntington disease can ameliorate the affects of the disease [67]. No protein aggregation has been reported in NPC, but an analogous mechanism could be at work: during the first several weeks of life, cells may activate autophagy to degrade the abnormal lipid-laden lysosomes and allow the survival of PCs. If this is true, then cells with lipid-laden lysosomes should have high autophagic activity. This prediction is clearly contradicted by our data: activation of autophagy is seen only in older, degenerating PCs, and *npc1* cells not undergoing cell death do not exhibit increased levels of the autophagic marker LC3-II.

Why then is autophagy induced in degenerating PCs? Autophagy is a normal, cellular process that is regulated by nutritional status and by hormones. Loss of *npc1* leads to trapping of lipids within aberrant membrane compartments, and this may induce a “lipid-starvation response” analogous to the well-characterized autophagic response to amino acid deprivation. In fact, there is already evidence that such a response occurs in *npc1* homozygotes: despite the seeming overabundance of cholesterol in *npc1^−/−^* cells, the SREBP signaling pathway is activated. This results in increased cholesterol synthesis and low density lipoprotein receptor activity [72]. Thus, NPC cells act as though they are “cholesterol starved.” NPC cells therefore may have three ways to adjust to a shortage of sterols and other lipids: increased lipid synthesis, increased lipid uptake, and autophagy. Microarray analysis of SREBP targets in liver [73] did not reveal increased transcription of autophagy genes, but the targets may differ between tissues, or regulation may occur at the post-transcriptional level. In NPC cells, the autophagic response would be futile since transport systems are damaged. Instead of rescuing the cell, autophagy would hasten cell death by further sequestering and reducing available lipids.

Destructive autophagy in NPC mutant PCs may also, or instead, be stimulated hormonally. In mammals, autophagy is inhibited by insulin [74], and in insects, steroids regulate autophagy of neurons by a cell-autonomous mechanism [75,76]. In *Manduca* (tobacco hawkmoth) motor neurons and in salivary glands and fat body of *Drosophila,* the steroid ecdysone triggers autophagy at specific developmental time points. We would infer a different regulatory relationship in mammalian NPC. Neurosteroids might inhibit autophagy in PCs and when their synthesis is severely decreased, as in NPC [61], autophagic cell death might ensue. Neurotrophins may also be involved in regulating PC autophagy. Cultured PCs undergo autophagic cell death following neurotrophin withdrawal [77]. These findings are relevant to NPC because neurons from NPC mice have decreased responsiveness to neurotrophins [78].

### Levels of Cerebellum Involvement in Chimeric *npc1^−/−^* Mice

The chimeric mice also provide information about the progression of the disease. Homozygous *npc1* mice at 70 d are unable to maintain posture on the balance beam for even a few seconds and weigh only half as much as controls. Remarkably, some chimeras had only minimal ataxia and no wasting despite the loss of approximately 40% of PCs. The minimal ataxia phenotype, manifested as occasional falls off the balance beam while turning, stopped progressing by approximately 140 d. By this age, nearly all mutant PCs, including those in lobule X, had died. Presumably ataxia worsens until all mutant PCs die, at which point the phenotype stabilizes.

With so many PCs lost, other regions of the brain, other cerebellar cells, or the remaining PCs may compensate. Morphological changes that have been observed in the remaining *npc1* neurons, such as occurrence of ectopic dendritic spines and meganeurites [26,79] and increased width of the dendritic tree [80], may be the physical manifestations of the remaining PCs taking on functions of their deceased counterparts. Compensation may also occur in other regions of the brain. In the *pcd* and *Lurcher* mouse models, compensatory mechanisms in the efferent targets of the PCs, the cerebellar deep nuclei, have been postulated to be responsible for the mild ataxia that occurs despite severe PC loss [81]. *Lurcher* chimeric mice, in which PCs undergo cell-autonomous loss, have no behavioral abnormalities even when they have few wild-type cells [82]. Recent studies using this model of PC degeneration have defined a minimum number of PCs required for normal motor control between 1,000–7,000, or roughly 1%–5% of the normal number of PCs [83,84]. Thus, the loss of PCs may be significantly ameliorated by still-to-be-identified mechanisms, and slowing loss of PCs by any mechanism may allow increased compensation. Some of the phenotypes of the *npc1^−/−^* mouse are likely to be due to loss or derangement of other cells, such as certain populations of neurons in the thalamus [85] or dorsal root ganglion [86]. There appears to be a critical threshold of wild-type contribution between 30% and 60% required for near-complete rescue of both ataxia and weight loss. The delayed progression seen with low levels of wild-type cells (approximately 15%) is quite encouraging with respect to the potential for therapies that preserve even some of the cells.

Several questions regarding the loss of PCs in NPC have been answered in this study. Of four possible mechanisms of PC loss (see Figure 1), the chimeric mice demonstrate that the two strictly non-cell-autonomous models are invalid. Of the two cell-autonomous models, internal toxicity versus subcellular deficiency, we favor the latter based on the observed defects in organelle trafficking in *npc1* cultured cells [87,88], the lack of correlation between cholesterol levels and PC loss, the decreased levels of steroidogenesis in *npc1* mice [61], and the previous transgenic studies designed to decrease cholesterol or sphingolipid accumulation [59,60]. We envision that in response to the proposed intracellular deficiency, cells compensate by increasing endogenous synthesis of cholesterol, by endocytosis, and/or by autophagy. In cells with a high lipid demand and insufficient supply, autophagy increases and cell death ensues. It may be that a similar series of events—defective trafficking leading to subcellular deficiency and autophagic cell death—occurs in many other lysosomal storage diseases. To our knowledge, Danon disease, caused by deficiency in the lysosomal membrane protein LAMP-2, is the only other lysosomal storage disease that has a demonstrated increase in AVs [89]. The pattern of degeneration erupting in each disease would be determined by the cell-specific response to being deprived of a particular molecule or class of molecules. PCs appear to be highly sensitive to insufficient levels of sterols or sterol metabolites (and perhaps also to sphingolipid deficiency, since PC loss is prominent in Niemann-Pick type A as well [90,91]).

Our results are particularly interesting in light of the two other human neurodegenerative diseases that have been analyzed with chimeric mouse models. In amyotrophic lateral sclerosis, surrounding wild-type non-neuronal cells can prevent the degeneration of *SOD1* mutant motor neurons, and the genotype of the motor neurons themselves appears to have no bearing on their probability of survival [23]. In the *mnd* mouse, a model for neuronal ceroid lipofuscinosis, proteolipid storage is cell-autonomous, but retinal degeneration equivalent to the *mnd* controls was seen in all chimeric mice [24]. The fact that three neurodegenerative diseases examined with this experimental approach exhibit three different modes of degeneration (non-cell-autonomous rescue for amyotrophic lateral sclerosis, cell-autonomous storage but non-cell-autonomous degeneration for neuronal ceroid lipofuscinosis, and cell-autonomous autophagic death for NPC) demonstrates the remarkable diversity in neuronal degeneration mechanisms and highlights how much still needs to be learned about the biology of neurodegeneration.

## Materials and Methods

### 

#### Materials.

BALB/c *npc1^nih^*, FVB.Cg-Tg(GFPU)5Nagy/J, and C57BL/6J mice were obtained from Jackson Laboratory (Bar Harbor, Maine, United States). CD-1 mice were from Charles River Laboratories (Wilmington, Massachusetts, United States). Mouse anti-Calbindin D-28K (1:250 dilution used), mouse anti-S100β (1:250) ascites, rapamycin, and filipin were obtained from Sigma (St. Louis, Missouri, United States). Rabbit anti-GABAα6 (1:250), rabbit anti-NeuN (1:100), and rat anti-myelin basic protein (1:500) were obtained from Chemicon International (Temecula, California, United States). Rat anti-F4/80 (1:100) was obtained from Serotec (Raleigh, North Carolina, United States). Mouse anti-CC1 (1:100) was from EMD Biosciences (San Diego, California, United States). Rabbit anti-MAP LC3 (1:1,000) was the kind gift of Marlene Rabinovitch and Lihua Ying (Stanford University, Palo Alto, California, United States). Nuclear dyes 7AAD and TOTO-3 were obtained from Molecular Probes (Eugene, Oregon, United States).

#### Generation of *npc1* chimeric mice.

Aggregation chimeras were made according to established procedures [92]. Mice made up of *npc1* cells and GFP-expressing wild-type cells were constructed using BALB/c *npc1^nih^* and FVB.Cg-Tg(GFPU)5Nagy/J embryos. In order to make reciprocal chimeras, *npc1* heterozygous mice were mated to FVB.Cg-Tg(GFPU)5Nagy/J mice. Offspring heterozygous for *npc1* and *GFP* were backcrossed to FVB.Cg-Tg(GFPU)5Nagy/J mice to obtain mice heterozygous for *npc1* and homozygous for *GFP*. Sibling matings were used to generate GFP-expressing *npc1^−/−^* embryos. Wild-type embryos were obtained from CD-1 and C57BL/6J strains. Week-old pups were examined with a blue LED and blue-filtering glasses to determine the ratio of mutant to wild-type cells based on skin fluorescence. The percentage of mutant cells in chimeras made with the C57BL/6J strain was also evaluated by measuring relative areas of coat color; the results were consistent with the percentage of GFP fluorescent cells.

Tail preparation DNA was used to genotype mice using PCR primers for *npc1* [6]. To distinguish between the wild-type *npc1* alleles in the BALB/c, FVB.Cg-Tg(GFPU)5Nagy/J, CD-1, and C57BL/6J strains, we took advantage of a polymorphism that could be detected with an ApaLI restriction digest. Digestion of the BALB/c, CD-1, or C57BL/6J wild-type PCR product results in 477-, 340-, and 130-bp fragments; the FVB.Cg-Tg(GFPU)5Nagy/J wild-type PCR product digest results in 817- and 130-bp fragments; the mutant *npc1* PCR product digest results in 937- and 130-bp fragments. Therefore, for *npc1^−/−^* ↔FVB.Cg-Tg(GFPU)5Nagy/J mice, we expect 937-, 817-, and 130-bp bands. For *npc1^−/−^*;GFP*^ +/+^*↔CD-1 or C57BL/6J mice, we expect 937-, 477-, 340-, and 130-bp bands.

#### Mouse assays.

Mice were weighed weekly on a gram scale. For balance beam measurements, mice were placed perpendicularly on a stainless steel bar (2 cm in diameter, 120 cm in length) wrapped in laboratory tape as described [29]. Alternating 10-cm segments were marked by varying tape color. The level of ataxia was assessed based on time (180 s maximum), number of sections crossed, and qualitatively on a five-point ataxia scale: five, no ataxia; four, inability to turn around on the bar; three, difficulty walking to the end of the bar without falling off; two, the mouse can only cling to the bar and is unable to correct itself from its initial perpendicular orientation; and one, postural instability as the mouse quickly falls off the bar even when placed along the long axis.

For gait assays, front paws were dipped in red paint and back paws in green paint, and mice were placed on Whatman paper at one end of a 14 × 44 cm box. The distance between the back edge of each same-side paw print was used to determine stride length.

#### Cerebellar sectioning and staining.

Mice were anesthetized with avertin and perfused with 4% PFA. Brains were post-fixed for 24 h in 4% PFA, transferred to 25% sucrose overnight, embedded and frozen in OCT, and cut into 12-μm sections with a cryostat.

For immunostaining, sections were rehydrated in PBS and blocked/permeabilized with 10% normal goat serum and 0.2% Triton X-100 in PBS. Incubation with primary antibody was carried out overnight at 4 °C. Following three washes with PBS, sections were incubated for 2–4 h with the appropriate fluorescently conjugated secondary antibody. Sections were washed again and mounted with Fluormount-G (Southern Biotechnology Associates, Birmingham, Alabama, United States). Filipin staining was carried out with 50 μg/ml filipin following treatment with 0.02% saponin as described previously [79].

#### PC counts.

All PCs in five sections at least 100 μm apart were counted for each mouse. The length of the PC layer was measured using MetaMorph software (Universal Imaging, Downington, Pennsylvania, United States), and the density was determined by dividing the number of PCs by this length. Expected densities if no degeneration has occurred were calculated by multiplying the percentage of chimerism by the density of PCs observed in *npc1* mice at 30 d. PC densities in the three chimeric mice sacrificed at 70 d were also compared to expected densities with degeneration (calculated from the percentage of chimerism and the density of PCs observed in *npc1* mice at 70 d). *p*-Values were calculated with the unpaired *t*-test or one-way ANOVA with Tukey's multiple comparison test using GraphPad Prism (GraphPad Software, San Diego, California, United States).

#### LC3 immunoblotting.

Cell extracts were obtained by solubilization with 2% IGEPAL-CA630, 0.2% SDS, and mini complete protease inhibitor tablet (Roche Applied Science, Indianapolis, Indiana, United States) in PBS, followed by centrifugation at 10,000 RPM for 10 min. Extracts were kept on ice and resolved with 15% SDS-PAGE. Following transfer to PVDF membrane (Bio-Rad, Hercules, California, United States), blocking was accomplished with 5% milk in PBS plus 0.1% Triton X-100. Primary incubation with anti-LC3 (rabbit, 1:1,000 in 1% milk PBST) was carried out overnight at 4 °C. Following three washes, the membrane was incubated with anti-rabbit-HRP (1:10,000), washed three times, and developed with WestPico reagent (Pierce Biotechnology, Rockford, Illinois, United States).

#### Electron microscopy.

Mice were anesthetized using avertin and were perfused with PBS followed by a solution of 2.5% glutaraldehyde and 4% paraformaldehyde in 0.1 M cacodylate buffer (pH 7.4) for 15 min using gravity flow. For microwave processing, a Pelco laboratory microwave equipped with variable wattage, Cold Spot water recirculator, and vacuum chamber was used (Ted Pella, Mountain Lakes, California, United States). Brains were removed, immersed in the same fixative, and microwaved at 100 W for 1 min on, 1 min off, 1 min on, with the Cold Spot set at 15 °C. A vacuum chamber was applied and the brains microwaved at 450 W for 20 s on, 20 s off, 20 s on, three times. After an additional 15 min at room temperature, the cerebellum was dissected away and sliced into anterior and posterior samples. Post-fixation was carried out using 2% osmium tetroxide containing 0.8% potassium ferricyanide and 5% sucrose in 0.1 M cacodylate buffer (pH 7.4) using the same microwave parameters as for primary fixation and followed by an additional 45 min at room temperature. After rinsing in distilled water, the tissue was dehydrated in an ascending alcohol (50%–95%) series at 350 W for 45 s, then switched to 100% ethanol under vacuum for 1 min on, 1 min off, 1 min on, three times. The tissue was microwaved in 100% acetone for 1 min on, 1 min off, 1 min on, at power level 3 using the vacuum chamber. The tissue was then infiltrated in Epon-Araldite-acetone mixture in the microwave for 4 min on, 4 min off, 4 min on, at 350 W for each resin mixture (1:2, 1:1, 2:1) under vacuum. For the final infiltration, the samples were placed in 100% resin and microwaved at 350 W under vacuum for 10 min on, 10 min off, 10 min on. The samples were embedded in fresh Epon-Araldite and hardened at 65 °C for 1–2 d. Semi-thin sections (1–2 μm) were cut using a Histoknife (Diatome, Fort Washington, Pennsylvania, United States) and stained with toludine blue. Thin sections (70 nm) were cut and mounted on formvar-coated multi-slot grids. Sections were post-stained with 5% aqueous uranyl acetate and lead citrate and examined at 80 kV in a JEOL (Tokyo, Japan) 1230 electron microscope. Digital images were captured with a Gatan (Pleasanton, California, United States) 967 slow-scan, cooled CCD camera. Images were analyzed with ImageJ Software (National Institutes of Health, Bethesda, Maryland, United States).

## Supporting Information

### Accession Numbers

The OMIM (http://www.ncbi.nlm.nih.gov/entrez/query.fcgi?db=OMIM) accession number for NPC is 257220. The NCBI Entrez (http://www.ncbi.nih.gov/entrez/query.fcgi?db=gene) accession numbers for the genes and gene products discussed in this paper are LC3 (GeneID 84557), *npc1* (GeneID 4864), and *npc2* (GeneID 10577).

## Abbreviations

AV: autophagic vacuole
C[number]: chimera [number]
CHO: Chinese hamster ovary
EM: electron microscopy
ER: endoplasmic reticulum
GFP: green fluorescent protein
LC3: light chain 3
NPC: Niemann-Pick type C
PC: Purkinje cell
