# Correction: Cell-Autonomous Death of Cerebellar Purkinje Neurons with Autophagy in Niemann-Pick Type C Disease

**DOI:** 10.1371/journal.pgen.0010086

**Published:** 2005-12-30

**Authors:** Dennis C Ko, Ljiljana Milenkovic, Steven M Beier, Hermogenes Manuel, JoAnn Buchanan, Matthew P Scott

In *PLoS Genetics,* volume 1, issue 1, DOI: 10.1371/journal.pgen.0010007

In the first paragraph of the Materials and Methods section, “rabbit anti-NeuN” should be “mouse anti-NeuN.”

